# Homojunction and defect synergy-mediated electron–hole separation for solar-driven mesoporous rutile/anatase TiO_2_ microsphere photocatalysts†

**DOI:** 10.1039/c9ra00633h

**Published:** 2019-03-08

**Authors:** Haoze Li, Bojing Sun, Fan Yang, Zhen Wang, Yachao Xu, Guohui Tian, Kai Pan, Baojiang Jiang, Wei Zhou

**Affiliations:** Key Laboratory of Functional Inorganic Material Chemistry, Ministry of Education of the People's Republic of China, Heilongjiang University Harbin 150080 P. R. China zwchem@hotmail.com

## Abstract

The photocatalytic hydrogen evolution of TiO_2_ is deemed to be one of the most promising ways of converting solar energy to chemical energy; however, it is a challenge to improve the photo-generated charge separation efficiency and enhance solar utilization. Herein, black mesoporous rutile/anatase TiO_2_ microspheres with a homojunction and surface defects have been successfully synthesized by an evaporation-induced self-assembly, solvothermal and high-temperature surface hydrogenation method. The H500-BMR/ATM (H*X*-BMR/ATM, where *X* means the different hydrogen calcination temperatures) materials not only possess a mesoporous structure and relatively high specific surface area of 39.2 m^2^ g^−1^, but also have a narrow bandgap (∼2.87 eV), which could extend the photoresponse to the visible light region. They exhibit high photocatalytic hydrogen production (6.4 mmol h^−1^ g^−1^), which is much higher (approximately 1.8 times) than that of pristine mesoporous rutile/anatase TiO_2_ microspheres (3.58 mmol h^−1^ g^−1^). This enhanced photocatalytic hydrogen production property is attributed to the synergistic effect of the homojunction and surface defects in improving efficient electron–hole separation and high utilization of solar light. This work proposes a new approach to improve the performance of photocatalytic hydrogen production and probably offers a new insight into fabricating other high-performance photocatalysts.

## Abstract 2

Mesoporous rutile/anatase TiO_2_ microspheres with surface defects are fabricated and exhibit excellent solar-driven photocatalytic performance due to synergistic effect of the homojunction and surface defects favoring efficient e–h separation.

## Graphical abstract

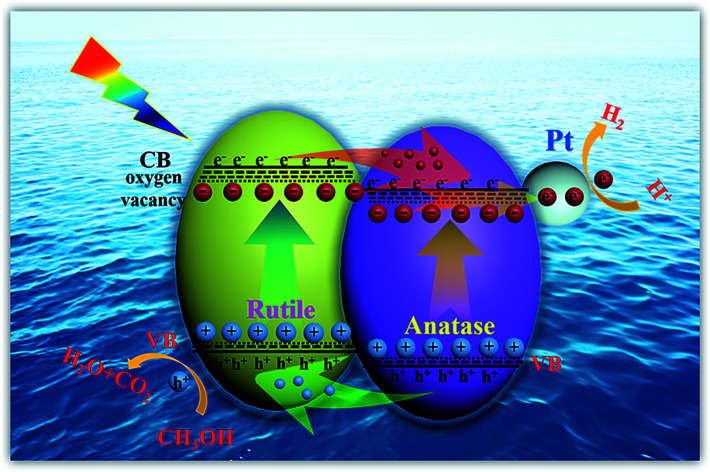

## 1 Introduction

The energy crisis and environmental pollution are two classic problems in today's world, which must be resolved in the near future.^1–3^ And new clean renewable energy sources play significant roles in addressing the solution of these two major problems.^4–8^ According to the literature, solar energy has attracted widespread attention, possessing the advantages of being clean, non-polluting, widely distributed, and abundant in reserves.^9–12^ However, solar energy is difficult to directly industrialize because it is not continuous and has unstable characteristics. Fortunately, converting solar energy into chemical energy can compensate for this problem, and it has high-energy-density, facilitating the virtues of energy storage and transportation. Among new energy sources, hydrogen energy has the advantages of being highly efficient and clean, produces no pollution, is easy to produce, is convenient for transportation and is renewable, which could make it the ideal energy carrier.^6,10,13,14^ Therefore, hydrogen energy has an increasingly important role in energy sources to replace fossil fuels. In recent years, semiconductor photocatalytic water splitting for hydrogen evolution technology has become a promising strategy because of its environmental friendliness, low-cost and cleanliness by using solar energy.

The titanium dioxide (TiO_2_) photocatalyst has been intensively investigated during the past several decades due to its cheapness, nontoxicity, and higher refractive index and stable physicochemical properties.^15–20^ Compared with single-phase TiO_2_, rutile/anatase mixed TiO_2_ (such as P25) enabled the transfer of electrons excited *via* solar light from rutile to anatase TiO_2_ because the energy of the anatase phase is slightly below the conduction band (CB), which can greatly suppress charge recombination and improve the photocatalytic performance.^21–25^ Previous studies have verified this result.^52^ However, although the rutile/anatase mixed phase of TiO_2_ can efficiently enhance its photocatalytic performance, TiO_2_ alone is limited to absorbing ultraviolet light, which covers less than 5% of the solar energy spectrum. Fortunately, Mao and co-workers reported a black TiO_2_ by a high-temperature surface hydrogenation method, which possessed a narrowed bandgap and extended photoresponse features.^26^ Since then, more and more researchers have been paying attention to the study of black TiO_2_, which has opened up a new era for TiO_2_ photocatalysis.^6,10,27–32^ Nevertheless, the most reported black TiO_2_ materials were generally nanoparticles, which were non-porous materials with a low surface area. Therefore, mesoporous black TiO_2_ is an ideal material for the efficient use of solar energy.

Here, we report a facile evaporation-induced self-assembly, solvothermal and high-temperature surface hydrogenation method to synthesize black mesoporous rutile/anatase TiO_2_ microspheres with a homojunction and surface defects. The as-prepared samples possess a narrow bandgap and wide-spectrum response, owing to the existence of surface defects and the homojunction. The semiconductor photocatalytic hydrogen production rate for H500-BMR/ATM is about 6.4 mmol h^−1^ g^−1^, which is approximately 1.8 times as high as that of mesoporous rutile/anatase TiO_2_ microspheres under air calcination (denoted MR/ATM ∼3.58 mmol h^−1^ g^−1^).

## 2 Experimental section

### 2.1 Chemicals

Titanium tetrabutoxide (TBOT), acetic acid (HOAc) and tetrahydrofuran (THF) were purchased from Shanghai Aladdin Bio-Chem Technology Co., Ltd. Hydrochloric acid (HCl) and ethanol were obtained from Tianjin Kemiou Chemical Reagent Co., Ltd. Pluronic F127 (PEO_106_PPO_70_PEO_106_, *M*_w_ = 12 600 g mol^−1^) was purchased from Sigma-Aldrich company. All chemicals were analytical reagents and used directly without any further processing.

### 2.2 Synthesis

The mesoporous rutile/anatase TiO_2_ microspheres were synthesized by a classical method. 1.6 g of F127 was completely dissolved in 30 mL of THF with vigorous stirring for 15 min, and then 0.2 mL of H_2_O, 2.4 mL of HOAc and 2.0 mL of HCl (36 wt%) were slowly added to the mixed reaction solution with drastic stirring for 30 min at normal temperature. Thereafter, 3.4 mL of TBOT was slowly mixed under magnetic stirring for 2 h to obtain a golden liquid. Subsequently, the obtained golden solution was transferred to a blast drying oven to evaporate THF solvent at 40 °C for 500 min. Afterwards, the golden solution was transferred to a 50 mL autoclave and reacted at 80 °C for 1400 min. After that, the obtained samples were washed 3 times with ethanol and distilled, respectively, and then dried at 80 °C overnight. Then the as-synthesized samples were calcined at 500 °C for 3 h under an air atmosphere. Finally, the white powder samples were calcined at 400, 500 and 600 °C for 3 h under a hydrogen atmosphere to obtain black mesoporous rutile/anatase TiO_2_ microspheres (denoted H*X*-BMR/ATM, where *X* means the different hydrogen calcination temperatures).

### 2.3 Characterization

The mesoporous rutile/anatase TiO_2_ microspheres were measured by X-ray diffraction over the diffraction angle range (2*θ*) 20–60° with a Bruker-Norius D8 advanced diffractometer, using a CuKα (*λ* = 1.5406 Å) radiation source operated at 40 kV and 40 mA. The images of transmission electron microscopy (TEM) were obtained with a JOEL JEM 2100F instrument operated at 200 kV. The pictures from scanning electron microscopy (SEM) were obtained with a Hitachi S-4800 instrument working at 15 kV. Diffuse reflectance spectroscopy (DRS) was measured by a UV/vis spectrophotometer (Shimadzu UV-2550) in the range of 200–800 nm. Nitrogen adsorption–desorption isotherms at 77 K were collected on an AUTOSORB-1 (Quantachrome Instruments) nitrogen adsorption apparatus. All samples were degassed under vacuum at 150 °C for at least 5 h prior to the measurement. The Brunauer–Emmett–Teller (BET) equation was used to calculate the specific surface area. Pore size distributions were obtained using the Barrett–Joyner–Halenda (BJH) method from the adsorption branch of the isotherms. Surface photovoltage spectroscopy (SPS) tests were carried out with a home-built apparatus equipped with a lock-in amplifier (SR830) synchronized with a light chopper (SR540). The photoluminescence (PL) spectra were studied by a PE LS 55 spectrofluoro-photometer with an excitation wavelength of 319 nm. A scanning Kelvin probe (SKP) test (SKP5050 system, Scotland) was executed to evaluate the work function at ambient atmosphere.

### 2.4 Photocatalytic activity

The photocatalytic hydrogen production tests were carried out in an on-line photochemical reaction system at room temperature (AuLight, Beijing, CEL-SPH2N). Classically, the photocatalysts (100 mg) were loaded with Pt (2 wt%) evenly dispersed in 80 mL of deionized water and 20 mL of methanol. The suspension of photocatalysts was sealed in the closed-gas circulation reaction cell. Prior to reaction, the reactor and the entire photocatalytic gas circulation system were degassed by a vacuum pump for about 40 min. Hydrogen evolution was studied under AM 1.5G irradiation with a power density of 100 mW cm^−2^ from a 300 W Xe lamp and was measured by an on-line gas chromatograph. The measurement of the apparent quantum efficiency (AQE) for hydrogen evolution was performed using the same closed circulating system under bandpass filter (365 and 420 nm) irradiation from a 300 W Xe lamp (SP7800, TCD, 5 Å molecular sieve, Ar carrier, Beijing Keruida Limited).

### 2.5 Photoelectrochemical test

Photoelectrochemical performances were measured using a Princeton Versa STAT 3 in a standard three electrode configuration with H500-BMR/AMT and MR/AMT materials used as photoanodes, Ag/AgCl reference electrode, Pt foil as the counter electrode. And 1 M KOH solution thoroughly purged with N_2_ for 60 min was used as the electrolyte. The photoanodes were prepared *via* a traditional spray coating method, using a glass rod to roll a paste containing 50 mg of the MR/ATM materials and 3 mL of ethanol on a transparent FTO glass-substrate with an effective diaphragm area of 1 cm^2^ (TCO, fluorine doped SnO_2_ layer, 20 Ω per square, Nippon sheet glass, Japan), followed by calcining 2 h at 350 °C under a N_2_ atmosphere, then calcining at 500 °C hydrogen temperature to obtain H500-BMR/ATM material. An AM 1.5 power system was used as the light source.

## 3 Results and discussion

**Fig. 1:**
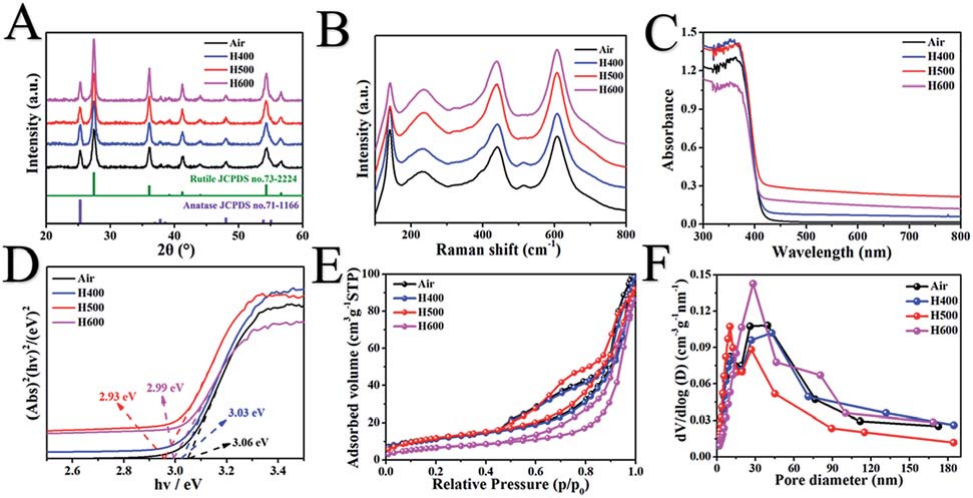
X-ray diffraction patterns (A), Raman spectra (B), UV/vis diffuse reflectance spectra (C), the corresponding optical bandgaps (D), N_2_ adsorption–desorption isotherms (E) and the corresponding pore size distribution plots (F) of mesoporous rutile/anatase TiO_2_ microspheres after calcination at different temperatures under a hydrogen atmosphere.

As shown in Fig. 1A, the X-ray diffraction (XRD) patterns of the MR/ATM samples show obvious diffraction peaks at 2*θ* = 27.3°, 36.1°, 39.1°, 41.3°, 44.0°, 54.3° and 56.7°, which can be indexed as (110), (111), (200), (111), (210), (211) and (220) indicating the rutile phase,^13,33,34^ and 2*θ* = 25.3° (101), 37.8° (004) and 48.1° (200) indicating the anatase phase,^6,9,10^ indicating that the as-prepared samples are rutile and anatase mixed phase. In addition, Fig. 1A shows that the peak intensity of the anatase phase gradually decreases and the rutile phase gradually increases with an increase in the hydrogenation temperature, which indicates that the content of the rutile phase increases with the increase in temperature, and the content of the anatase phase gradually decreases, which can be seen in Table S1.† In order to obtain better confirmation of the structural changes of TiO_2_, Raman spectroscopy was also conducted. The peaks at 143, 437 and 610 cm^−1^ can be assigned to the classical vibrational of B_1g_, E_g_ and A_1g_, which are characteristic of the rutile phase.^33,35,36^ In addition, the peak at 238 cm^−1^ is caused by the multi-proton scattering process. It is worth pointing out that the Raman peaks at 513 cm^−1^ can be attributed to the typical anatase bands of A_1g_ (B_1g_), further confirming that the mesoporous TiO_2_ microspheres are comprised of the rutile/anatase phase. Another interesting finding is that the peak at 513 cm^−1^ is gradually weakened as the hydrogenation temperature increases, indicating that the content of the anatase phase gradually decreases with increasing hydrogenation temperature. The result is in good agreement with the XRD analysis. The UV/vis diffuse reflectance in Fig. 1C shows an obvious absorption peak at wavelengths less than 400 nm, which can be assigned to the innate band gap absorption of rutile/anatase TiO_2_. Meanwhile, the visible-light absorption is gradually enhanced with an increase in the hydrogen calcination temperature, owing to the production of more surface defects.^37^ The surface disorder layer after hydrogenation of rutile/anatase TiO_2_ can form mid-gap states, which is beneficial to visible-light absorption. As shown in Fig. 1D, the bandgap of the as-prepared samples is reduced with the increase in hydrogen calcination temperature, further confirming there are more surface defects.^38^ And the bandgap of H500-BMR/ATM is only 2.93 eV, which is beneficial for visible light photocatalysis.

In order to obtain structural information on the pore structure and Brunauer–Emmett–Teller (BET) specific area, N_2_ adsorption–desorption analysis on the calcined samples was performed. Fig. 1E and F show the adsorption–desorption isotherms and BJH pore size distribution curves of MR/ATM at different hydrogen calcination temperatures. As can be seen, the adsorption–desorption isotherms are typical type IV curves, indicating mesoporous materials.^6,13^ Until the hydrogen temperature is increased to 500 °C, the adsorption–desorption curves show almost no significant change, indicating that the mesoporous structure can still be effectively and stably retained. When the hydrogen calcination temperature continues to increase to 600 °C, the adsorption–desorption isotherm changes, indicating that the structure of the sample calcined at this temperature has been changed, which is probably caused by partial pore collapse. The pore size distribution curve shows that the H*X*-BMR/ATM material has a narrow pore distribution, indicating that the MR/ATM material possesses relatively uniform pores, and the pore diameter is approximately 15 nm. The surface areas (as shown in Table S2†) of the as-synthesized MR/ATM, H400-BMR/ATM, H500-BMR/ATM and H600-BMR/ATM are estimated to be 52.6, 44.1, 39.2 and 20.3 m^2^ g^−1^, respectively. The pore volumes of MR/ATM, H400-BMR/ATM, H500-BMR/ATM and H600-BMR/ATM are measured to be 0.14, 0.14, 0.13 and 0.11 cm^3^ g^−1^, respectively. The result might be owing to the rising hydrogen calcination temperature causing partial tunnel collapse, shrinkage and particle agglomeration.

**Fig. 2:**
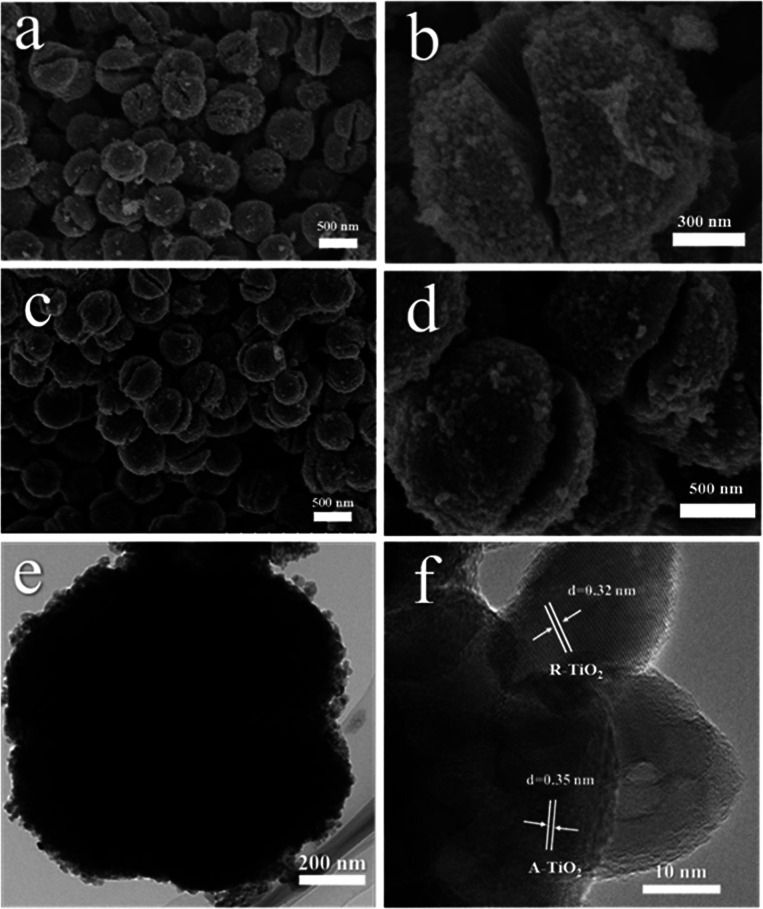
SEM images (a and b) of MR/ATM, and SEM (c and d), TEM (e), HETEM (f) images of H500-BMR/ATM.

The morphology of the as-synthetized mesoporous microspheres possessed a dehiscent configuration according to the scanning electron microscopy (SEM), as shown in Fig. 2a–c. In addition, it can be clearly observed that the diameter of the mesoporous microspheres is 1.2 μm on average, and the distribution is particularly uniform. It is worth mentioning that the morphology of the hydrogenated sample did not change significantly compared to the pristine one, which indicates that the as-prepared samples possess high thermal stability. A typical transmission electron microscopy (TEM) image, as presented in Fig. 2c, indicated mesoporous microspheres with a diameter of about 1.2 μm, which coincided with the SEM observation. As shown in Fig. 2d, high-resolution TEM (HRTEM) was used to characterize the H500-BMR/ATM sample. The H500-BMR/ATM sample shows a characteristic lattice spacing of 0.35 nm, which corresponds to the (101) lattice planes of anatase and the expected lattice spacing of 0.32 nm, which matches well with the (110) planes of rutile.^13,33,39–42^ The results indicate that the as-prepared samples are a mixed phase of rutile and anatase, which coincides with the XRD and Raman analysis.

**Fig. 3:**
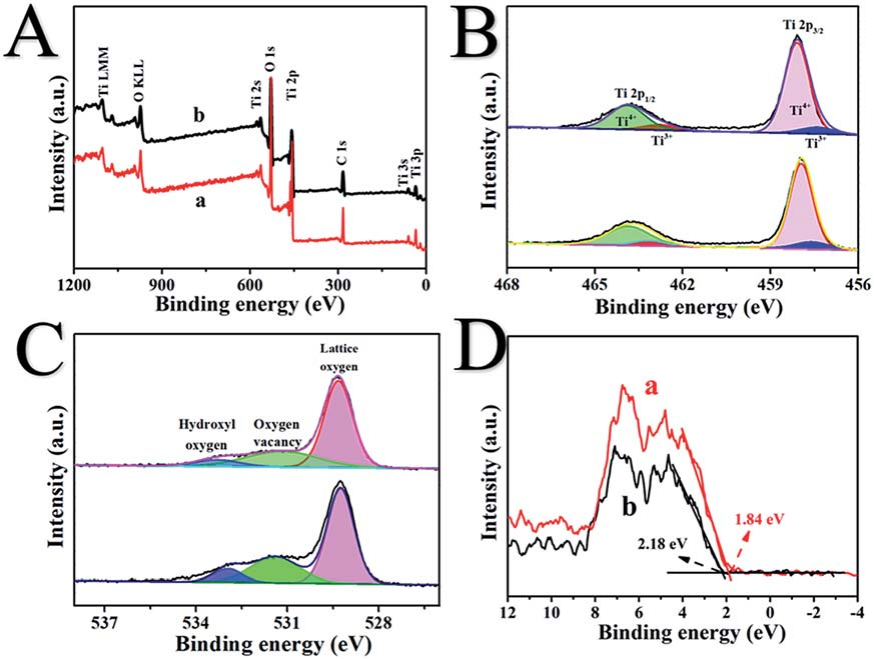
The full-scale XPS spectra (A), Ti 2p (B), O 1s (C) and valence band XPS (D) of H500-BMR/ATM (a) and MR/ATM (b), respectively.

Surface structures of the as-synthetized samples were measured by X-ray photoelectron spectroscopy, as shown in Fig. 3. Fig. 3A reveals that in the full spectrum of H500-BMR/ATM and MR/ATM, we can clearly observe the presence Ti 3s, Ti 3p, and O 1s peaks, indicating that the obtained sample is a pure TiO_2_ material. And other peaks are not found with calcination by high-temperature hydrogen, indicating that the sample structure was stable. Fig. 3B shows the Ti 2p spectra of H500-BMR/ATM and MR/ATM. The peaks located at 458.1 eV and 463.9 eV are attributed to Ti 2p_3/2_ and Ti 2p_1/2_ orbitals for Ti^4+^, respectively.^29,31^ And the other two peaks located above at 457.2 eV and 462.9 eV are derived from Ti 2p_3/2_ and Ti 2p_1/2_ peaks for Ti^3+^. Moreover, it can be clearly seen that the Ti^3+^ peaks of the H500-BMR/ATM samples are significantly enhanced compared with the MR/ATM samples, indicating the effect of high-temperature hydrogenation.^37^ The O 1s spectra show three separated peaks (Fig. 3C). The two peaks at 529.3 and 533.0 eV coincide with lattice oxygen and hydroxyl oxygen, respectively. The peak at 531.4 eV can be ascribed to the oxygen vacancy generated by surface hydrogenation.^43^ Moreover, the peak intensity of the H500-BMR/ATM material at 531.4 eV is much higher than that of the MR/ATM material, indicating that the sample after high-temperature hydrogenation possesses more oxygen vacancy defects, which can improve the separation efficiency of electrons and holes. As shown in Fig. 3D, the XPS valence band (VB) of H500-BMR/ATM and MR/ATM can be observed. The VB of H500-BMR/ATM is located at ∼1.84 eV, which is more negative than that of MR/ATM (∼2.18 eV), indicating that surface hydrogenation significantly increases the VB and obviously reduces the bandgap. As we all know, electron paramagnetic resonance (EPR) technology is considered to be one of the most effective methods for characterizing the presence of oxygen vacancies in metal oxides. As shown in Fig. S1,† an obvious sharp resonance signal of H500-BMR/ATM at *g* = 2.002 can be clearly observed, which can be attributed to the electron trapped in an oxygen vacancy.^50,51^ However, the peak of the sample MR/ATM at *g* = 2.002 is not very obvious, indicating that the sample H500-BMR/ATM hydrogenated by the high-temperature surface has a higher number of oxygen vacancies. This result is completely consistent with the spectral results of O 1s in XPS.

**Fig. 4:**
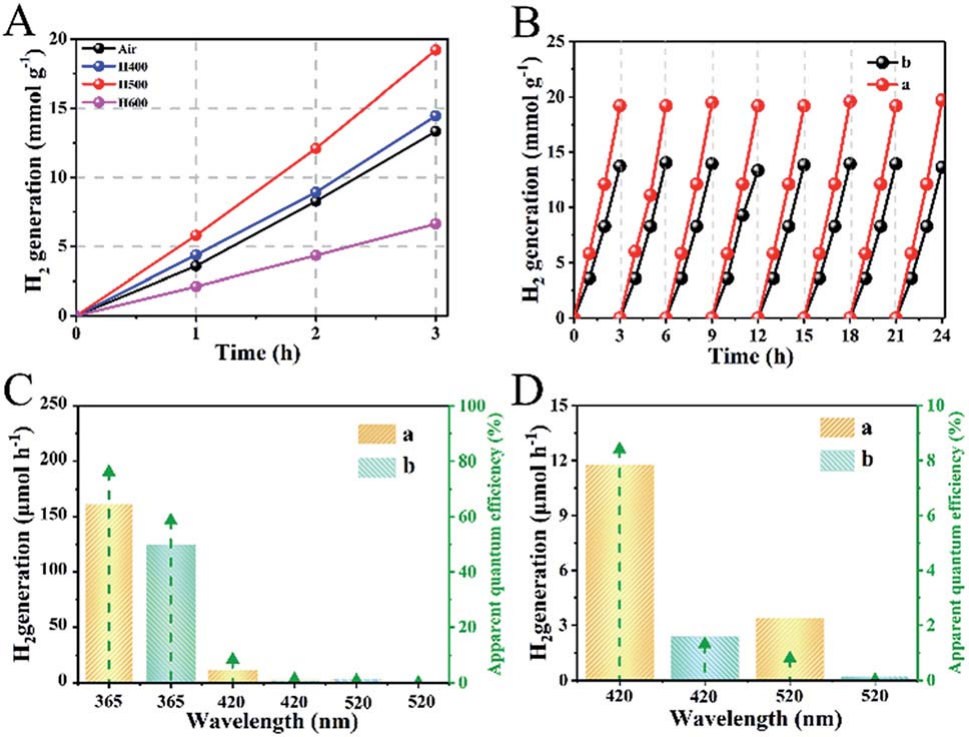
Photocatalytic hydrogen generation rate (A) by TiO_2_ calcined at different temperatures, cycling tests of photocatalytic hydrogen generation (B), the single-wavelength photocatalytic hydrogen evolution rate and the corresponding apparent quantum efficiency (AQE) (C) and AQE for amplifying single-wavelength light at 420 and 520 nm (D) of H500-BMR/ATM (a) and MR/ATM (b), respectively.

In Fig. 4A, all of the as-prepared samples exhibited excellent photocatalytic hydrogen evolution performance under AM 1.5 illumination. In particular, the as-prepared sample of H500-BMR/ATM can produce H_2_ at 6.4 mmol h^−1^, much higher than the other samples with different hydrogen calcined temperatures, even almost 1.8 times higher than that of MR/ATM (3.58 mmol h^−1^). In order to further confirm the stability of the photocatalytic hydrogenation performance, cycle experiments were carried out within 24 h. After testing 8 cycles, the amount of photocatalytic hydrogen production did not decrease significantly, as shown in Fig. 4B, further determining the high stability of all the as-prepared samples. Furthermore, an experiment to test for photocatalytic hydrogen production was also performed under single wavelength irradiation (Fig. 4C). Clearly, the hydrogen generation rate of H500-BMR/ATM is 162.2 μmol h^−1^ at 365 nm, which is better than the performances at 420 nm (11.8 μmol h^−1^) and 520 nm (3.4 μmol h^−1^) (Fig. 4D), which means that the main reason for the high photocatalytic hydrogen performance is the excitation of electrons for water splitting under ultraviolet light illumination. As a control, MR/ATM produces much less hydrogen at each wavelength than that of H500-BMR/ATM material, further indicating that the hydrogenation process can enhance photocatalytic activity. In addition, according to formula (1),^44,45^ the apparent quantum efficiency (AQE) for different single wavelengths is 76.2, 8.4 and 0.8 at 365, 420 and 520 nm, respectively. The result exhibits that the mesoporous rutile/anatase TiO_2_ microspheres after hydrogenation manifestation have excellent visible light photocatalytic activity.1
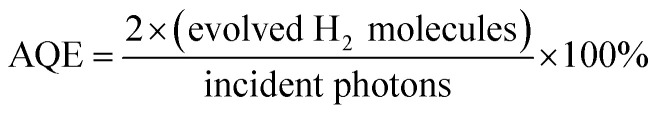


**Fig. 5:**
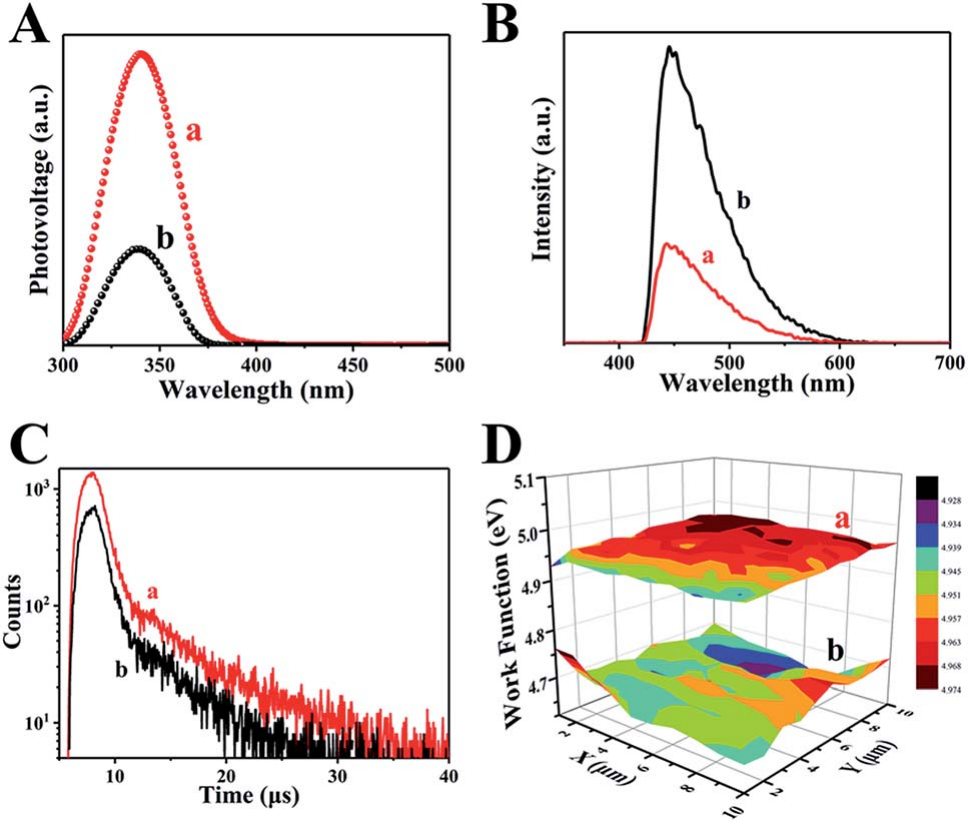
The surface photovoltage spectroscopy (A), transient-state fluorescence spectrum (B), fluorescence spectra (C) and the scanning Kelvin probe results (D) of H500-BMR/ATM and MR/ATM, respectively.

The separation of photogenerated electron–hole pairs is analyzed by surface photovoltage spectroscopy (SPS).^45^ As shown in Fig. 5A, a strong SPS peak at approximately 340 nm for H500-BMA/RTM and MA/RTM can obviously be observed, owing to the electron transitions from the valence band (VB) to the conduction band (CB). In addition, the SPS peak intensity of the H500-BMA/RTM material is much higher than that of the MA/RTM material, indicating the higher photogenerated carrier separation efficiency and longer excitation lifetime for the former. It should be pointed out that the sample of H500-BMA/RTM showed a significant red-shift, further indicating an improvement in the visible light photocatalytic activity. The photoluminescence (PL) spectra also exhibit the behaviour of separation of photogenerated electrons and holes, as they mainly study the reorganization of photogenerated electrons and holes.^46^ As shown in Fig. 5B, a strong peak at nearly 450 nm can be clearly seen, owing to the electron transitions from the VB to the CB. Compared to H500-BMR/ATM, the PL intensity of MR/ATM is much higher, indicating that the photogenerated electrons and holes have a higher probability of recombination, further demonstrating that H500-BMR/ATM is more detrimental to photocatalytic performance. The test results of PL are completely consistent with the SPS results. In addition, the transient-state fluorescence spectra were measured to study the photogenerated charge carrier lifetime (Fig. 5C).^47^ The result indicates that the photogenerated charge carrier lifetime of H500-BMR/ATM (13.86 μs) is much longer than that of MR/ATM (11.59 μs), which proves that the hydrogenation process helps to enhance the separation of photogenerated charge carriers. The scanning Kelvin probe (SKP) is a measurement technique of vibration-capacitance-based non-contact lossless gas phase metal surface potential, which is used to measure the material's work function or surface potential.^48^ The work functions of H500-BMR/ATM and MR/ATM are ∼4.96 and ∼4.69 eV, respectively. This indicates that the Fermi level of H500-BMR/ATM is slightly lower than that of MR/ATM, and slightly detrimental to photocatalytic performance. However, the existence of rich surface defects on H500-BMR/ATM provides more active sites, which accelerates the transfer of photoelectrons to the surface, thereby greatly improving the separation efficiency of electrons and holes, thereby enhancing the photocatalytic hydrogenation performances.

**Fig. 6:**
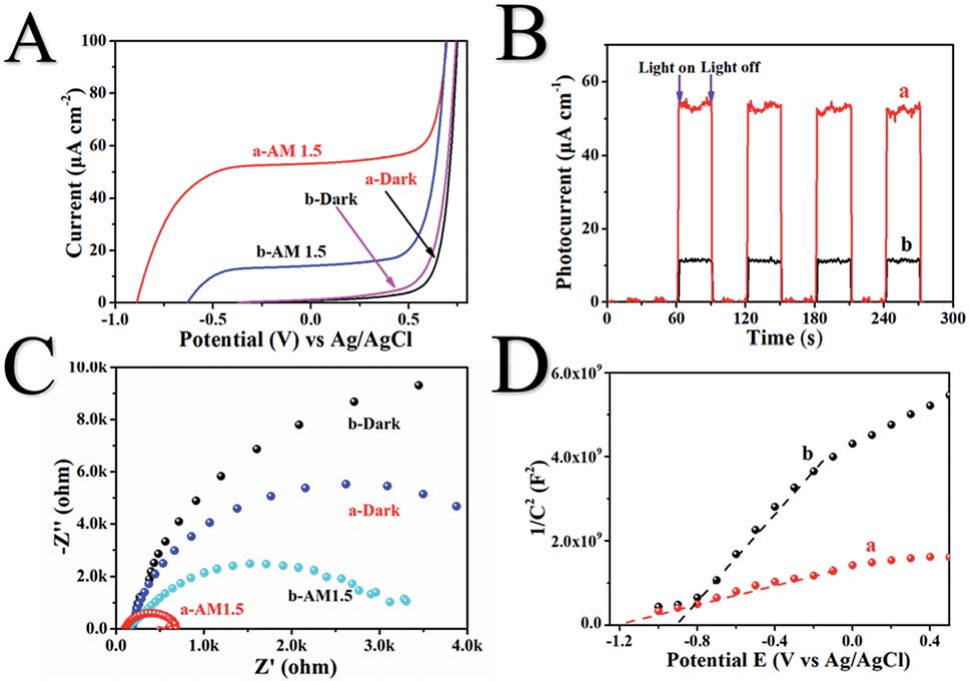
The linear sweep voltammograms (A), chronoamperometry (B), Nyquist plots of electrochemical impedance spectroscopy (C) and Mott–Schottky plots (D) of H500-BMR/ATM (a) and MR/ATM (b), respectively.

The photoelectrochemical properties of H500-BMR/ATM and MR/ATM materials are tested by a Princeton VersaSTAT. The linear sweep voltammograms (LSV) in Fig. 4A show that H500-BMR/ATM possesses a higher current density (52.2 μA cm^−1^) than Air-MR/ATM (14.3 μA cm^−1^) under AM 1.5 illumination, which exhibits a more conducive charge separation. In addition, both samples exhibited low current density under dark conditions, illustrating the higher separation efficiency of photogenerated electron–hole pairs under irradiation conditions. Furthermore, the transient chronoamperometry on the samples of H500-BMR/ATM and MR/ATM is shown in Fig. 6B, indicating that both samples possess high photoelectrochemical stability. Both H500-BMR/ATM and MR/ATM materials exhibit prompt and constant light-on and -off responses under AM 1.5 illumination. The electrochemical impedance (EIS) measurements display that the interfacial resistance of H500-BMR/ATM is smaller than that of MR/ATM, demonstrating that the H500-BMR/ATM material possesses more efficient charge mobility. Notably, when the test conditions are in the dark, both H500-BMR/ATM and MR/ATM materials show large interfacial resistance, indicating that the charge transfer can be facilitated by AM 1.5 illumination. The Mott–Schottky (M–S) plots (Fig. 6D) show that both H500-BMR/ATM and MR/ATM samples have positive slopes, exhibiting that all materials are n-type semiconductors. Moreover, the material of H500-BMR/ATM exhibits a small slope compared with that of MR/AM, indicating that the former possesses a higher charge carrier density. The increased charge carrier density for H500-BMR/ATM could be owing to the presence of Ti^3+^ and surface oxygen vacancy defects, which are beneficial to improving the electron–hole pair transport and separation, furthermore enhancing the photocatalytic activity.

**Scheme 1:**
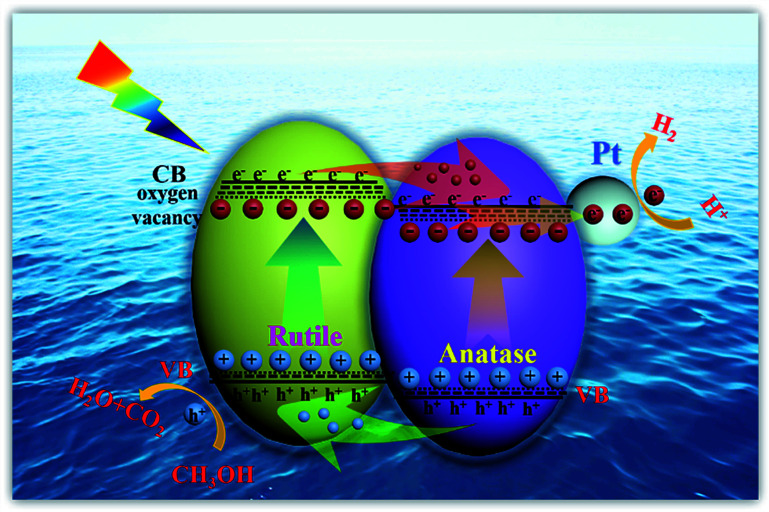
Schematic illustration of homojunction and surface defect mediated photogenerated electron–hole separation in TiO_2_ photocatalytic hydrogen evolution.

Herein, the excellent photocatalytic hydrogen evolution property of H500-BMR/ATM can be ascribed to the following reasons. Firstly, the homojunction effect between rutile and anatase favours the separation of photogenerated electron–hole pairs and is an important reason behind the improved photocatalytic activity. As we all know, rutile phase TiO_2_ has a slightly lower energy gap than anatase phase TiO_2_ (3.02 eV *vs.* 3.20 eV). When rutile and anatase phases are combined, a staggered band gap is formed and the homojunction effect can lead to an effective charge separation across the phase junctions (Scheme 1). Most researchers believe that under solar excitation, photogenerated electrons are transferred from rutile to anatase and holes flow in the opposite direction. Photogenerated electrons can be moved from the CB of rutile to the CB of anatase and further transfer to surface sites of metal Pt.^49^ Finally, the electrons on metal Pt will reduce the protons to produce hydrogen, further increasing the photocatalytic hydrogen production activity. Another reason is that owing to the calcination by a high-temperature hydrogen atmosphere, surface defects of oxygen vacancies and Ti^3+^ are formed, causing the formation of a narrow bandgap and more mid-gap states. The surface oxygen vacancies are beneficial to the adsorption and dissociation of water molecules, making hydrogen molecules easy to generate. The synergistic effect of Ti^3+^, surface oxygen vacancy defects and the homojunction are favourable to the absorption of solar light, the rapid separation of photon-generated carriers, and the suppression of a rapid recombination rate, thereby improving the solar-driven photocatalytic hydrogen evolution property.

## 4 Conclusions

In summary, we have demonstrated a facile method to prepare a black mesoporous rutile/anatase TiO_2_ microsphere material possessing a homojunction and surface defects by an evaporation-induced self-assembly, solvothermal and high-temperature surface hydrogenation method. The H500-BMR/AMT sample exhibited excellent photocatalytic hydrogen evolution performance with a hydrogen production rate of 6.40 mmol h^−1^, which was ∼1.8 times higher than that of MR/ATM (∼3.58 mmol h^−1^). The results suggested that the existence of the homojunction and surface defects caused a narrowed bandgap and improved the separation efficiency of photogenerated electron–hole pairs, further enhancing the photocatalytic hydrogen evolution performance.

## Conflicts of interest

There are no conflicts to declare.

## Supplementary Material

RA-009-C9RA00633H-s001
