# Supplementary material for: Homojunction and defect synergy-mediated electron–hole separation for solar-driven mesoporous rutile/anatase TiO2 microsphere photocatalysts
Source: RSC Adv. 2019 Mar 8;9(14):7870–7. doi: 10.1039/c9ra00633h (PMC9061422; doi:10.1039/c9ra00633h)
Supplement: RA-009-C9RA00633H-s001 [file RA-009-C9RA00633H-s001.pdf]

Electronic Supplementary Material (ESI) for RSC Advances.
This journal is © The Royal Society of Chemistry 2019

Supporting Information

Homojunction and Defects Synergy-Mediated Electron-Hole Separation for Solar-Driven Mesoporous Rutile/Anatase TiO_2 Microsphere Photocatalysts

Haoze Li, Bojing Sun, Fan Yang, Zhen Wang, Yachao Xu, Guohui Tian, Kai Pan, Baojiang Jiang and Wei Zhou*

Key Laboratory of Functional Inorganic Material Chemistry, Ministry of Education of the People's Republic of China, Heilongjiang University, Harbin 150080, P. R. China

Email: zwchem@hotmail.com

Table S1. Ratio of rutile to anatase of the mesoporous rutile/anatase TiO_2 microspheres after being calcined at different temperatures under hydrogen atmosphere.

	Air	H400	H500	H600
Anatase (%)	27.5	22.9	16.2	15.2
Rutile (%)	72.5	77.1	83.8	84.8

Table S2. The textual properties of the mesoporous rutile/anatase TiO_2 microspheres after being calcined at different temperatures under hydrogen atmosphere.

	Air	H400	H500	H600
Surface Area (m^2 g^{-1})	52.6	44.1	39.2	20.3
Pore Size (nm)	13.8	12.5	12.2	16.5
Pore Volume (cm^3 g^{-1})	0.14	0.14	0.13	0.11

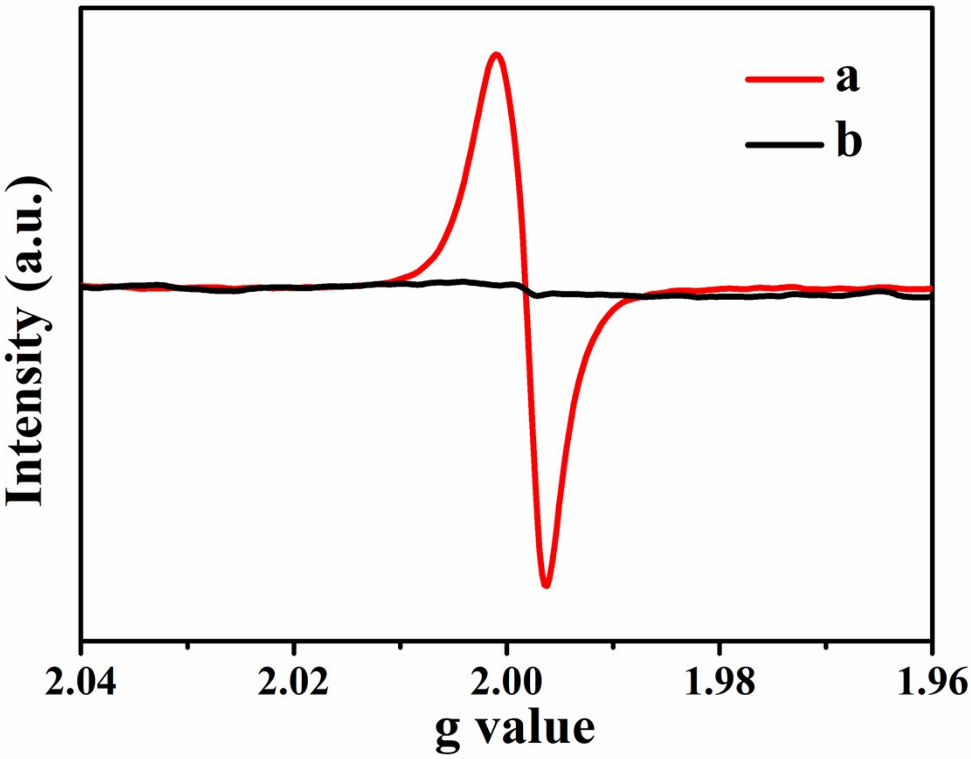

Figure S1. The electron paramagnetic resonance (EPR) spectra of MR/ATM (b) and H500-BMR/ATM (a).
